# Are thermal barriers "higher" in deep sea turtle nests?

**DOI:** 10.1371/journal.pone.0177256

**Published:** 2017-05-18

**Authors:** Pilar Santidrián Tomillo, Luis Fonseca, Frank V. Paladino, James R. Spotila, Daniel Oro

**Affiliations:** 1 Population Ecology Group, Institut Mediterrani d’ Estudis Avançats, IMEDEA (CSIC-UIB), Miquel Marquès, 21, Esporles, Mallorca, Spain; 2 The Leatherback Trust, Goldring-Gund Marine Biology Station, Playa Grande, Costa Rica; 3 Biocenosis Marina, Trinidad de Moravia, San José, Costa Rica; 4 Department of Biology, Indiana-Purdue University, Fort Wayne, Indiana, United States of America; 5 Department of Biodiversity, Earth and Environmental Science, Drexel University, Philadelphia, Pennsylvania, United States of America; Deakin University, AUSTRALIA

## Abstract

Thermal tolerances are affected by the range of temperatures that species encounter in their habitat. Daniel Janzen hypothesized in his “Why mountain passes are higher in the tropics” that temperature gradients were effective barriers to animal movements where climatic uniformity was high. Sea turtles bury their eggs providing some thermal stability that varies with depth. We assessed the relationship between thermal uniformity and thermal tolerance in nests of three species of sea turtles. We considered that barriers were “high” when small thermal changes had comparatively large effects and “low” when the effects were small. Mean temperature was lower and fluctuated less in species that dig deeper nests. Thermal barriers were comparatively “higher” in leatherback turtle (*Dermochelys coriacea*) nests, which were the deepest, as embryo mortality increased at lower “high” temperatures than in olive ridley (*Lepidochelys olivacea*) and green turtle (*Chelonia mydas*) nests. Sea turtles have temperature-dependent sex determination (TSD) and embryo mortality increased as temperature approached the upper end of the transitional range of temperatures (TRT) that produces both sexes (temperature producing 100% female offspring) in leatherback and olive ridley turtles. As thermal barriers are “higher” in some species than in others, the effects of climate warming on embryo mortality is likely to vary among sea turtles. Population resilience to climate warming may also depend on the balance between temperatures that produce female offspring and those that reduce embryo survival.

## Introduction

Understanding the mechanisms behind thermal tolerances is critical for assessing potential responses of animal populations to anthropogenic climate warming [1, 2]. Animal thermal tolerances, especially in ectotherms, are related to the temperatures experienced in the habitat and are broader in those areas of higher climatic variability [3, 4].

In his 1967 seminal article Janzen hypothesized that animal thermal tolerances were lower where climatic uniformity was high [5]. In his “Why mountain passes are higher in the tropics”, Janzen [5] hypothesized that topographic barriers were more effective at impeding animal distributions when climatic uniformity was high. He further proposed that it was temperature gradient and not absolute height across a mountain range that determined the effectiveness of the barrier [5], making mountain passes physiological barriers to animal dispersal. Although scientific discussions related to this central idea have primarily focused on the context of latitudinal variations in temperature and its effects on animal thermal tolerances [3, 6], it can also be applied to other barriers such as precipitation [7] and different life-history traits [8]. High and low temperatures can decrease survivorship of animal populations as species survival is constrained within specific thermal limits and the breadth of thermal tolerances depends on the range of temperature that species encounter in their natural habitat [3]. As a result, species inhabiting tropical areas are expected to be more sensitive to warming events than those in temperate sites, even after small changes, because tropical species are constrained by narrower temperature ranges [9]. Thus, they may not have been exposed to the selective forces to evolve physiological mechanisms to cope with large fluctuations or changes in the temperatures they experience.

Temperature gradients are not only found across mountain passes, but exist in practically all ecosystems and also occur underground. Thermal gradients in the soil at a particular site vary with air temperature, depth, water content and soil characteristics [10]. The selection of the nest site is particularly important in egg-burying reptiles, as it will determine the conditions that the clutch will experience during development [11] and eggs can only develop within certain thermal limits [10, 11].

Sea turtles bury their eggs in tropical, subtropical or temperate beaches. Depth of the nest depends on size of the female and on sand and beach characteristics, such as thickness of the surface layer of dry sand [12]. In general, bigger species dig deeper nests [13] and as a result, the thermal environment that developing clutches experience may vary among species. Since sand temperatures increase in stability with depth [14, 15], deeper nests experience more uniform conditions during development. Consequently, clutches laid by large species that naturally encounter more uniform thermal conditions may be more susceptible to changes in temperature, than those that are naturally exposed to higher fluctuations. In this study, we consider thermal barriers as physiological barriers to embryo survival. This concept gives an idea of the relative impact of temperature in the nest environment. Following Janzen’s study [5], thermal barriers are “high” when a small change in temperature has a large effect.

Temperature in the nest affects hatching success, emergence rate of hatchlings [16–18] and sex of hatchlings since sea turtles have temperature-dependent sex determination (TSD) [19]. Anthropogenic climate warming may affect sea turtles in various ways. Rising temperatures could (1) reduce hatchling output [18, 20, 21] eventually resulting in population declines [21, 22], (2) increase feminization since female hatchlings are produced at high temperatures [23, 24], and (3) reduce spatial availability of nesting sites due to sea level rise [23, 25]. Additionally, the effects of climate warming on sea turtles at sea could include changes in phenology, reproductive frequency, migration patterns and feeding conditions [22–24].

In the present study, we analyzed the relationship between thermal conditions in the nest environment and egg survival in three species of sea turtles. We hypothesized that sea turtle embryos could encounter thermal barriers in the nest and that these barriers are “higher” for species that dig their nests to greater depths, because thermal stability increases with depth. Thus, small changes in nest temperature would have a larger impact on egg mortality in large species whose clutches are placed deeper in the sand. As overall mean temperature is likely to increase throughout the 21^st^ century [26], higher temperatures due to climate warming may become impassable evolutionary barriers for those species that are most sensitive to changes in temperature.

## Materials and methods

**Fig 1:**
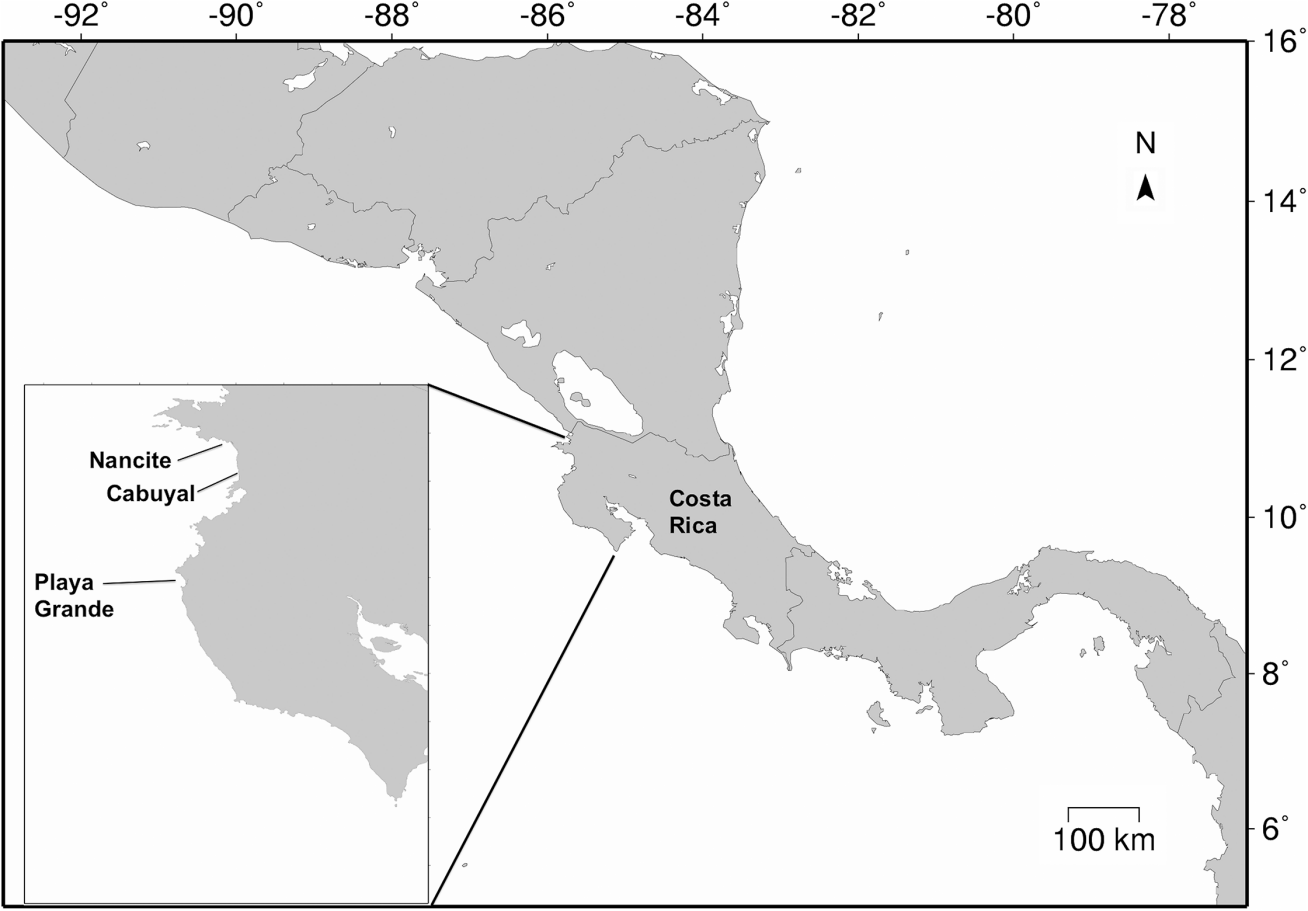
Location of study sites in Northwest Costa Rica. Nancite, Cabuyal and Playa Grande serve as nesting grounds for olive ridley, green and leatherback turtles respectively.

The Animal Care Committee of Drexel University approved the study and research permits were granted by the Ministry of Environment and Energy of Costa Rica. We compared the conditions in the nest environment of three sea turtle species, leatherback turtles (*Dermochelys coriacea*), green turtles (*Chelonia mydas*) and olive ridley turtles (*Lepidochelys olivacea*) that nest in the same general area in Northwest Costa Rica and dig their nests at different depths due to differences in body size [13]. North Pacific Costa Rica is a tropical area highly influenced by El Niño Southern Oscillation (ENSO) where previous studies have shown that climatic conditions influence the nest environment of leatherback turtles [17, 27]. We collected data on leatherback turtle, green turtle and olive ridley turtle nests from Playa Grande, Cabuyal and Nancite beaches respectively (Fig 1). These beaches were located within a maximum distance of 54 km from each other. Leatherback turtles are the largest sea turtle species [28] and have a mean curved carapace length (CCL) in the eastern Pacific of 147.0 cm [29]. East Pacific green and olive ridley turtles have mean CCLs of 86.2 cm [30] and 65.9 cm [31] respectively at the study sites. Mean nest depths are related to turtle size and at the study sites depths are 82.2, 68.1 and 47.3 cm respectively for leatherback, green and olive ridley turtle nests. We measured nest depth from the bottom of the egg chamber to the sand surface with a stick meter. Depth was obtained from a subsample of nests (Table 1).

**Table 1:** Hatching success and thermal conditions of sea turtle nests in North Pacific Costa Rica.

Species	Number nests	Nesting strategy	Hatching success	Mean temperature (°C)	Daily fluctuation (°C)	Seasonal fluctuation (°C)	Nest depth (cm) *
olive ridley	40	arribada	0.07 ± 0.17	34.2 ± 2.3	0.66 ± 0.39	6.7 ± 3.7	-
olive ridley	78	solitary	0.48 ± 0.41	32.5 ± 1.7	1.27 ± 1.38	7.1 ± 5.4	47.3 ± 7.6
green	78	-	0.87 ± 0.19	31.2 ± 1.2	0.21 ± 0.12	5.7 ± 2.2	68.1 ± 9.0
leatherback	985*	-	0.45 ± 0.29	31.0 ± 1.1	0.07 ± 0.02	5.1 ± 2.6	82.2 ± 8.3

Mean (± SD) hatching success, mean (± SD) temperature (°C), mean (± SD) daily fluctuation in temperature (°C), mean (± SD) seasonal fluctuation in temperature (°C) and mean (± SD) depth in olive ridley, green and leatherback turtle nests.

(*)N = 985 nests for estimations of mean hatching success, mean temperature and seasonal fluctuation in temperature and N = 5 nests for daily fluctuation in temperature in leatherback turtles.

Sample sizes for nest depth of olive ridley, green and leatherback turtles were 43, 62 and 514 nests respectively.

To monitor temperatures in leatherback nests, we used 24 gauge Cu–Cn thermocouples (www.omega.com) that were read with a Bat 12 thermocouple reader (±0.1°C) in seasons 2004–2005 to 2014–2015 (Table 2). In 2004–2005, we also used HOBO temperature Pro v2 data logger (±0.2°C) (http://www.onsetcomp.com) (Table 2). We programed data loggers to collect data hourly throughout the incubation period (mean incubation period at the study site is 59.9 days [32]). The long-term project at Playa Grande uses thermocouples and collects information once every other day in the afternoon (15:00–16:00 h). We use thermocouples instead of data loggers because thermocouples are accurate (±0.1°C) and inexpensive. One measurement per day is representative of daily temperature due the low variability in temperature at nest depth in leatherback turtles [17]. We used the detailed hourly information from the data loggers to estimate daily fluctuations in temperature and the thermocouple data for all other estimations because the dataset was much larger as it included 11 seasons.

**Table 2:** Nesting beaches where we obtained data on nest temperatures and hatching success per season. Information from leatherback, green and olive ridley turtles was collected at Playa Grande (PG), Cabuyal (CAB) and Nancite (NAN) respectively. Nest temperatures were monitored with thermocouples and/or dataloggers.

Season	Temperature (thermocouples)	Temperature (loggers)	Hatching success
2004–2005	PG	PG	PG
2005–2006	PG	-	PG
2006–2007	PG	-	PG
2007–2008	PG	NAN	PG, NAN
2008–2009	PG	-	PG
2009–2010	PG	NAN	PG, NAN
2010–2011	PG	NAN	PG, NAN
2011–2012	PG	CAB	PG, CAB
2012–2013	PG	CAB	PG, CAB
2013–2014	PG	CAB	PG, CAB
2014–2015	PG	CAB	PG, CAB

We used HOBO 8K Pendant temperature data loggers (±0.5°C) in green turtle (n = 78) and olive ridley turtle (n = 118) nests that we retrieved when we excavated nests after hatchlings had emerged. We obtained data on temperatures during nesting seasons 2011–2012, 2012–2013, 2013–2014 and 2014–2015 for green turtles and for nesting seasons 2007–2008, 2009–2010 and 2010–2011 for olive ridleys (Table 2). In all cases, we placed thermocouples or data loggers approximately in the middle of the clutch after ~50% of the eggs were interred and remained untouched throughout incubation. To characterize the thermal conditions in the nest environment, we calculated mean temperature during development, mean daily fluctuation and mean seasonal fluctuation in temperature for each nest.

Olive ridley turtles exhibit two different nesting strategies at Nancite. They nest in either mass-nesting events known as “arribada” or as solitary nesters [33]. Since the nest environment may differ between strategies, we classified nests as either “arribada” or “solitary” and we analyzed them in two manners, first pooled all together and then separately by nesting strategy. We had temperature data from arribada nests in 2009–2010 and from solitary nests in 2007–2008, 2009–2010 and 2010–2011. When making comparisons between species we only used solitary nests for olive ridley turtles (Table 1).

We excavated nests after hatchlings emerged at different times depending on the project. We estimated hatching success (*H*) following previous methodology, using the formula *H = S / (S + U)*, where *S* is number of hatched shells and *U* number of dead unhatched eggs [27]. We only included in situ nests in the study.

We compared nest temperature and hatching success for those species for which we had data for the same years. We made comparisons between leatherback and green turtles for seasons 2011–2012, 2012–2013, 2013–2014 and 2014–2015, and between leatherback and olive ridley turtles for seasons 2009–2010 and 2010–2011.

We used generalized additive models (GAM) to examine the non-linear relationship between mean incubation temperature (°C) and hatching success. GAMs have been previously used to test the relationship between mean incubation temperature and emergence success in the leatherback turtles of Playa Grande [17]. Smoothing splines produced a smooth generalization of the relationship between the two variables (nest temperature and hatching success) in a scatterplot for visual examination. We generated scatterplots for the three species for visual comparison of the relationship between nest temperature and hatching success.

We used the mgcv library [34] in R [35], version 3.0.1 for GAM and used SPSS v. 23 [36] for other analyses. We used SEATURTLE.ORG Maptool [37] to generate the map in Fig 1.

## Results

### Thermal conditions in the nest environment

**Fig 2:**
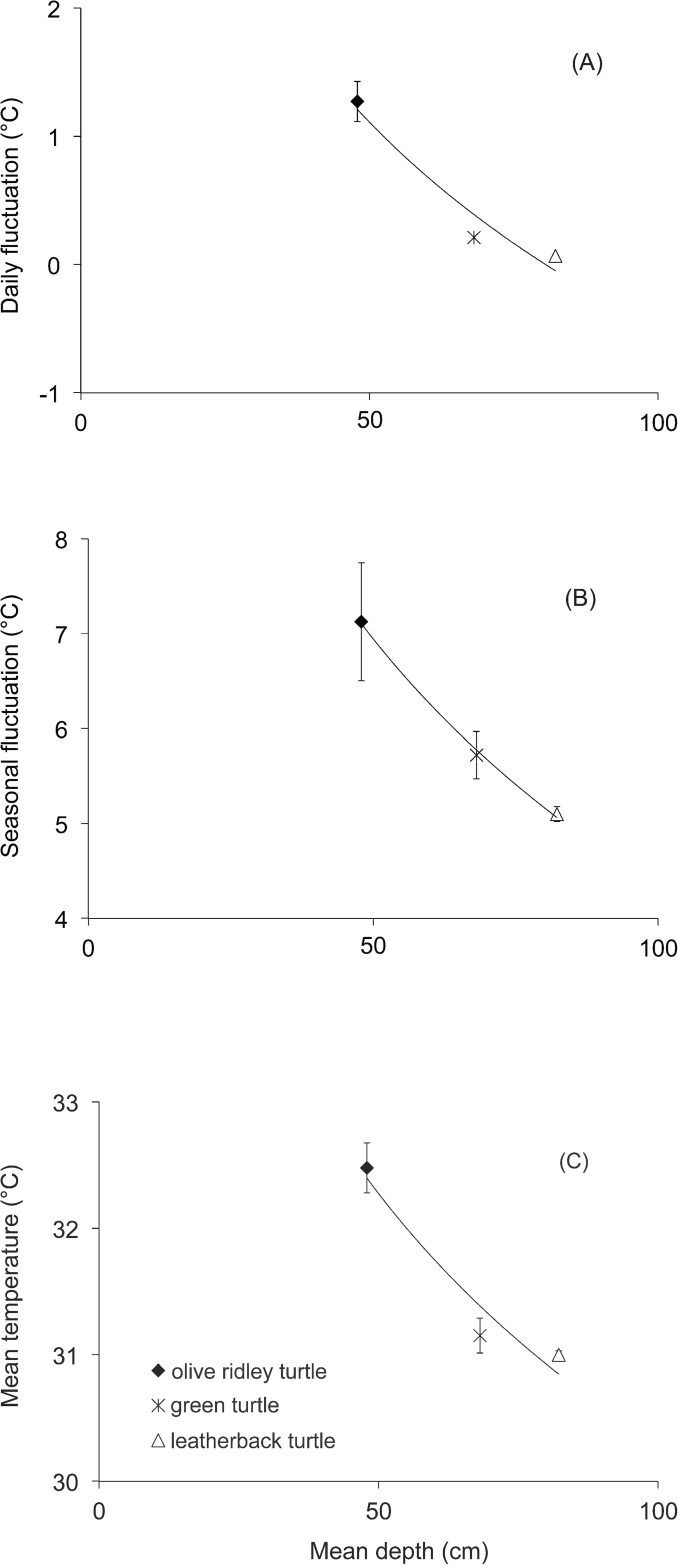
Thermal conditions in olive ridley, green and leatherback turtle clutches versus nest depth. (a) mean (± SE) daily fluctuation in temperature (°C), (b) mean (± SE) seasonal fluctuation (°C) and (c) mean (± SE) temperature (°C) during development. Regression line corresponds to a logarithmic fit.

Thermal stability increased with nest depth. Species-specific daily fluctuation and seasonal fluctuation of temperatures significantly decreased as the species-specific nest depth increased (logistic regression, *P* < 0.05 in both cases) (Fig 2). The species-specific mean temperature also decreased as nest depth increased but the relationship was not significant (P > 0.05) (Fig 2). There were no significant differences in mean temperature (Mann-Whitney U-test, *P* > 0.05) and in seasonal fluctuation of temperature (Mann-Whitney U-test, *P* > 0.05) between leatherback and green turtle nests. However, there were significant differences in mean temperature and seasonal fluctuation of temperature between olive ridley and leatherback nests (Mann-Whitney U-test, *P* < 0.001 both cases).

### Effect of temperature on hatching success

Green turtle clutches had a significantly higher hatching success than leatherback turtle clutches (U Mann-Whitney, *P* < 0.001). However, we found no significant differences in hatching success between leatherback and solitary olive ridley clutches (U Mann-Whitney, *P* > 0.05). There were significant differences in mean hatching success (U Mann-Whitney, *P* < 0.001), mean temperature (U Mann-Whitney, *P* < 0.001) and daily fluctuations in temperature (U Mann-Whitney, *P* < 0.001), but not in seasonal fluctuation (U Mann-Whitney, *P* > 0.05) between olive ridley clutches that were laid in arribadas and those laid during solitary nesting events. Mean temperature was higher and hatching success was lower in arribada nests when compared to solitary ones (Table 1).

**Fig 3:**
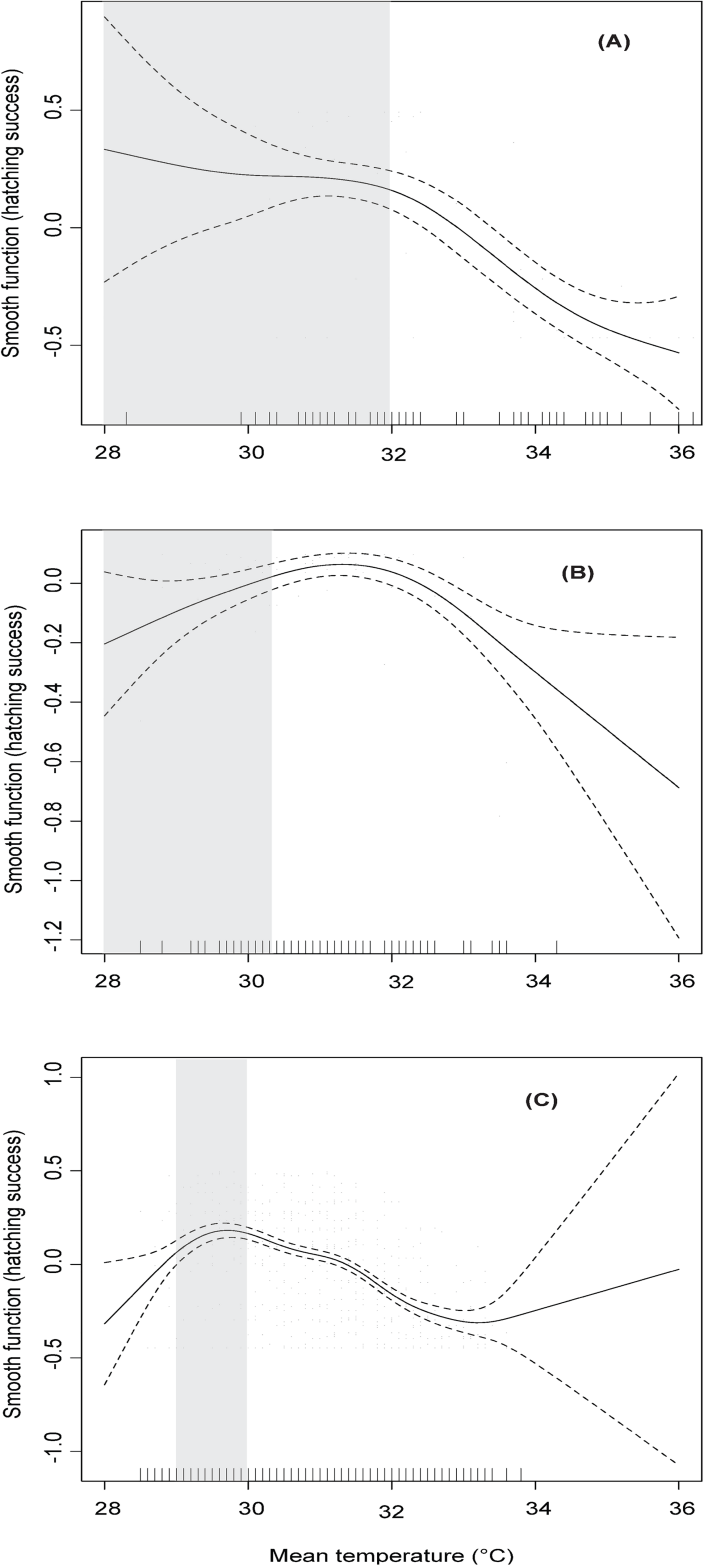
Smooth fits from generalized additive models (GAM) showing additive effect of nest temperature (°C) on hatching success. (a) olive ridley turtle, (b) green turtle and (c) leatherback turtle nests. Discontinued lines show two standard errors around main effect. Tick marks on the x-axis represents observed data points. Gray area shows the Transitional Range of Temperatures for sex determination for the leatherback [38] and olive ridley [39] populations based on published records.

**Fig 4:**
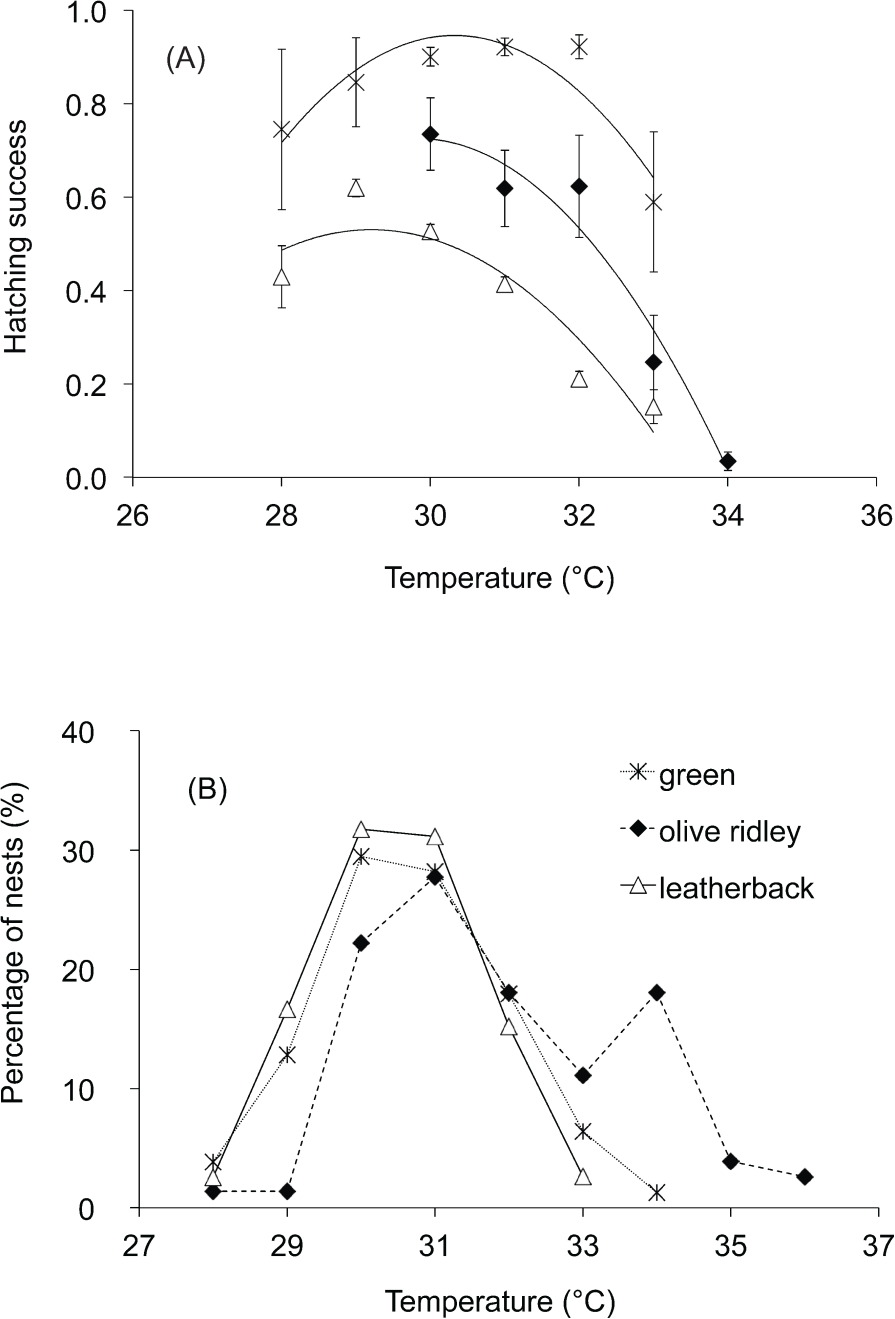
Mean hatching success and percentage of clutches of each species versus mean temperature (°C). (a) Mean hatching success of olive ridley, green and leatherback turtle clutches per mean temperature (°C) by 1 (°C) increments and (b) percentage of clutches per mean temperature (°C) by 1 (°C) increments. Regression line in Fig 4a corresponds to a polynomial fit.

Hatching success was significantly affected by mean temperature and declined as temperatures increased in the three species (GAMs: adjusted *R*^*2*^ = 0.43, 0.22 and 0.25 for olive ridley, green and leatherback turtles respectively, *P* < 0.001 all cases, Fig 3). High nest temperatures had the greatest negative effect on leatherback clutches as embryo mortality increased at lower “high” temperatures in leatherback turtles (~ 30°C) than in olive ridley and green turtles (both ~32°C). Green turtles had the highest and leatherback turtles had the lowest hatching success at each particular mean temperature (by 1°C increments, Fig 4, S1 Fig).

The temperature at which hatching success started to decline in leatherback (~ 30°C) and olive ridley (~32°) turtles coincided approximately with the temperature of the upper limit of their TRTs based on published records [38, 39], when offspring ratio reaches 100% female (Fig 3). Temperature-dependent sex determination has not been studied on the green turtle population that nest at Cabuyal and their TRT is unknown. The data showed in the GAM charts suggested that low temperatures could also have a negative effect on hatching success but the confidence intervals were wider at that end (Fig 3) due to low sample size, since less than 1%, 4% and 3% of nests were exposed to temperatures lower than 29°C during development in olive ridley, green and leatherback turtles respectively (Fig 4).

## Discussion

Our study suggests that thermal barriers exist in the nest environment of sea turtles and that there are differences in thermal tolerances among species. As expected, mean nest temperatures were lower and fluctuated less in species that nested deeper. The effect of high temperatures was also greater in leatherback turtles than in green and olive ridley turtles, as mean hatching success was lower and started to decline at lower temperatures. This suggests that the relationship between climatic uniformity and thermal barriers also applies to the nest environment. Leatherback turtles exhibit the lowest hatching success among sea turtles (~ 50%, [40]), but this varies globally as it is influenced by local climatic conditions [41]. In this study, we compared clutches of species that nest in the same area and therefore, were exposed to similar climatic conditions. However, the conditions in the nest environment still varied with depth making thermal barriers higher in the deepest leatherback nests.

Differences in thermal tolerances are not only found among species but also intra-specifically and within populations at fine scales [42, 43]. For example, clutches laid by green turtles that nest on a dark beach of Ascension Island where temperatures are naturally high exhibit higher thermal tolerance than those laid by turtles that use a nearby lighter beach [42]. Two conditions seem essential to develop specific thermal adaptations within a population. First, thermal conditions in the nest environment must differ between the nesting beaches and second, nest-site fidelity must be high. At the individual level, turtles that disperse their clutches over areas that have different thermal conditions may be more resilient to changes in temperature than those that consistently nest on the same beach and experience narrow thermal ranges. Nest-site fidelity in sea turtles is generally high and turtles tend to lay clutches in close proximity to previous ones [44, 45]. However, there are often individuals that nest on beaches over several hundred km apart [13]. While distributing nests spatially is a common behavior in sea turtles, the number of females in a population with poor nest-site fidelity is typically low [13]. Thus, inter-clutch variability in thermal conditions is probably low in most individuals.

Recent work suggests that under climate change (1) increasing seasonal temperature variability due to higher frequency of extreme events and (2) decreasing daily fluctuation in temperature due to differences in warming rates between day and night temperatures will have a greater impact on species with narrow thermal tolerances [46] such as the leatherback turtle. Adult leatherback turtles tolerate wide ranges of temperatures in the ocean [47], but the developing eggs may not adapt nor acclimate well to wide fluctuations in temperature even seasonally, due to the deep nest stability impacts on the evolution of these mechanisms responding to temperature.

Sea turtles have long generation times [48] so thermal tolerances must have developed over a long time period. Increased nest temperature due to climate warming constitutes an abrupt change under evolutionary time scales in long-lived species [22]. Population responses to climate warming at a particular site may depend on the (1) absolute temperatures projected for the site, (2) rate of change that could or could not allow adaptation and (3) vulnerability to changes in temperature. As the interest in modeling the impact of climate change on sea turtles grows, population projections should incorporate, not only climatic projections, but also population-specific mortality curves in relation to temperature, as well as other vital rates [49].

Olive ridley turtles experienced higher and more fluctuating temperatures than leatherback turtle clutches but there were no significant differences in hatching success between them. On the other hand, we found differences in hatching success between leatherback and green turtle clutches, but no differences in the thermal conditions of the nest. Nancite is an arribada beach with high levels of egg failure associated with arribadas because of higher nest-densities [33]. High nest density during arribadas results in decreased O_2_ and increased CO_2_ levels in the nest, as well as high microbial activity, which can reduce hatching success [50, 51]. Although nest-density is low between the arribadas at Nancite, the organic content and microbial levels in the sand are likely higher here than at any other solitary beach, which could still reduce hatching success. Additionally, olive ridley and leatherback nests may suffer from hydric stress [41]. These species nest on the open beach, as opposed to green turtles that tend to select vegetated areas that shade the nests [30, 52]. As a result, olive ridley and leatherback clutches may be more sun-exposed and water content at nest depth must be lower. Dry conditions in the nest environment can exacerbate the effect of temperature in leatherback nests in dry areas [40] and could also contribute to lowering hatching success of olive ridley nests. Additionally, ~ 45% of solitary clutches of olive ridleys included in the analysis were laid in season 2009–2010, when the conditions were especially dry and warm due to an El Niño event. In fact, all clutches that had mean temperatures equal or greater than 33.5°C, were laid in 2009–2010, which explains the high percentage of clutches that had mean temperatures between 34°C and 35°C (Fig 4B).

Mean hatching success of green turtles (0.87 ± 0.19) at Cabuyal was high and only declined at temperatures greater than 32°C. High hatching success has been previously reported for other populations of green turtles [53, 54] and the species may tolerate high temperatures well [55]. Embryo tolerance to high temperature was also reported for flatback turtles (*Natator depressus*) [28]. Although some populations may tolerate high temperatures better than others, the effect of climate warming on them will depend on temperature rise. All sea turtles have upper thermal limits [13]. Thus, declines are expected if temperatures rise over the specific temperature that increases embryo mortality.

Hatching success in leatherback and olive ridley turtles started to decline at the ~100% female producing temperatures. TSD may be adaptive in sea turtles as 100% female ratio is produced when embryo mortality increases, which mitigates the negative effect of temperature on the population growth [21] by increasing future fecundity (number of nesting females). However, once rising temperatures have passed the upper end of the TRT, populations will likely decline from reduced hatching success, as percentage of female offspring can no longer be increased [21]. Thus, population resilience to climate warming may also depend on the balance between temperatures that produce female offspring and those that reduce embryo survival. Highly female-biased sex ratios are not concerning under current conditions because operational sex ratios are balanced, at least in some populations [56]. However, that could change under extreme scenarios of climate change as embryo mortality continues to increase [21, 56].

Leatherback turtles have one of the narrowest TRT among reptiles [57, 58] and narrow TRTs have been associated with high frequency of unisexual clutches [59]. Additionally, species with narrow TRT, such as leatherback turtles and tuataras, may be more vulnerable to climate warming [20, 60] than others as they may not be able to adapt primary sex ratios quickly enough to sudden changes in temperature. Thus, abrupt changes in temperature may not only increase mortality in the nest, but also approach 100% female production in the clutches of turtles that dig deeper.

Although this is just a study and may not represent the mean, the ability of green turtles to tolerate higher temperatures compared to other sea turtles and its relationship with sex determination deserves further exploration. The number of nesting female green turtles has increased in several parts of the World, which has been attributed to successful implementation of conservation actions [61–63]. However, the number of nesting females could have also grown due to rising temperatures [64]. Female offspring ratio (and future fecundity) may have increased in recent decades due to rising temperatures, but mortality in the nest may have not yet increased [21]. Green turtles seem more resilient to increased temperatures than other sea turtles, but their populations could follow the trends projected for other species as the warming trend continues.

## Supporting information

S1 Fig Normalized mean hatching success versus mean temperature (°C). Mean hatching success of green, olive ridley and leatherback turtle clutches versus mean incubation temperature (°C) by 1 (°C) increments. Data were normalized at 30°C to facilitate comparison between the three species.
(PDF)
