# Supplementary material for: Are thermal barriers "higher" in deep sea turtle nests?
Source: PLoS One. 2017 May 18;12(5):e0177256. doi: 10.1371/journal.pone.0177256 (PMC5436680; doi:10.1371/journal.pone.0177256)

S1 Fig. Normalized mean hatching success versus mean temperature (°C). Mean hatching success of green, olive ridley and leatherback turtle clutches versus mean incubation temperature (°C) by 1 (°C) increments. Data were normalized at 30 °C to facilitate comparison between the three species.

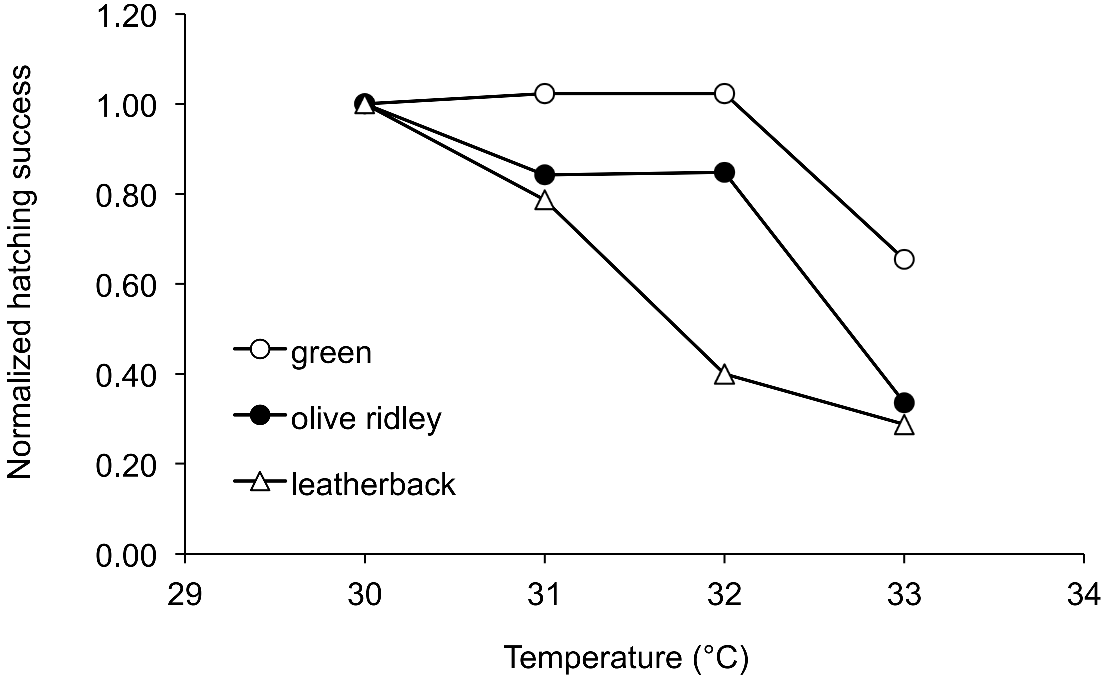

Supplement: S1 Fig — Mean hatching success of green, olive ridley and leatherback turtle clutches versus mean incubation temperature (°C) by 1 (°C) increments. Data were normalized at 30°C to facilitate comparison between the three species. (PDF) [file pone.0177256.s001.pdf]
